# Effects of a Pedometer-Based Walking Program in Patients with COPD—A Pilot Study

**DOI:** 10.3390/medicina58040490

**Published:** 2022-03-29

**Authors:** Yen-Huey Chen, Li-Rong Chen, Ching-Ching Tsao, Yu-Cheng Chen, Chung-Chi Huang

**Affiliations:** 1 Department of Respiratory Therapy, College of Medicine, Chang Gung University, Taoyuan City 33302, Taiwan; cch4848@adm.cgmh.org.tw; 2 Division of Pulmonary and Critical Care Medicine, Chang Gung Memorial Hospital, Linko, Taoyuan City 33353, Taiwan; 3 Department of Respiratory Care, Chang Gung University of Science and Technology, Chiayi 61363, Taiwan; 4 Master Degree Program in Healthcare Industry, College of Medicine, Chang Gung University, Taoyuan City 33302, Taiwan; lena5114@gmail.com; 5 Division of Pulmonary and Critical Care Medicine, Department of Internal Medicine, Taoyuan General Hospital, Taoyuan City 33004, Taiwan; n002286@gmail.com (C.-C.T.); chenxanzai@gmail.com (Y.-C.C.)

**Keywords:** COPD, pedometer, physical activity, quality of life

## Abstract

*Background and objectives:* Patients with chronic obstructive pulmonary disease (COPD) suffer from impaired pulmonary function and dyspnea, which result in limited levels of physical activity, and impaired quality of life. Exercise and regular physical activity have been proven to break the vicious circle. The aim of this pilot study is to investigate the effects of a walking program on exercise capacity and quality of life in patients with COPD. *Materials and Methods:* Patients with COPD were randomly assigned to a pedometer group (PG) or control group (CON). Subjects in the PG walked target steps daily with a pedometer for six weeks. Before and after the program, the following measurements were performed: pulmonary function test (PFT), daily steps, Six-Minute Walk Test (6 MWT), COPD Assessment Test (CAT), and quality of life questionnaire (SF-12). *Results:* After this walking program, PG (*n* = 15) significantly improved their daily steps from 4768.4 ± 2643.3 steps to 7042.7 ± 4281.9 steps (*p* = 0.01). Forced vital capacity (FVC) increased from 2.5 ± 0.7 L to 2.8 ± 0.9 L (*p* = 0.02). CAT scores decreased from 14.9 ± 8.8 points to 11.5 ± 7.5 points (*p* = 0.03). In the control group (*n* = 11), there were no differences in any outcomes after this daily walking program. *Conclusions:* For patients with COPD, a daily walking program with a pedometer is beneficial in the improvement of pulmonary function, daily steps, and quality of life.

## 1. Introduction

Chronic obstructive pulmonary disease (COPD), a leading cause of mortality and morbidity worldwide, is projected to rise from 5.4 million in 2005 to 8.3 million in 2030 [1,2,3]. In the United States, COPD was the third most common cause of death and the only disease among the top 10 that continued to increase in prevalence [4,5]. An international guideline states that regular physical activity is beneficial for COPD patients by improving symptoms, functional independence, and quality of life [1].

However, physical inactivity is a major clinical feature of COPD. As the disease progresses, COPD causes breathlessness, fatigue, and exercise intolerance, resulting in the reduction of physical activity levels. Decreased physical activity levels are often associated with poor lung function, muscle strength decline, impaired quality of life, and frequency of hospitalization [6,7].

Interventions to increase the physical activity level of COPD patients are essential for the improvement of prognosis. Inpatient and/or outpatient pulmonary rehabilitation programs have been proven to improve exercise capacity and quality of life in patients with COPD [8]. Guidelines for the management of COPD have suggested that pulmonary rehabilitation including exercise should be provided [8]. However, there are barriers for COPD patients participating in hospital-based pulmonary rehabilitation programs, such as transportation requirements, geographic distance from the hospital, and adherence to the pulmonary rehabilitation program [9,10]. Thus, there is a need to find a convenient and effective intervention to promote physical activity for COPD.

Walking is a common physical activity and is essential for the independence of performing activities in daily life. Daily steps and time spent on the steps have been suggested as indicators of functional capacity [11]. Pedometers are easy to use and can provide feedback to individuals about their daily activities. Studies showed that pedometers are an effective tool to monitor and increase physical activity in healthy populations [12]. However, it is unclear whether the use of pedometers has similar benefits in COPD populations. The primary purpose of this study was to examine the effects of a pedometer-based home walking program on the levels of physical activity. The secondary outcome was to determine its effects on pulmonary function, symptoms, and quality of life.

## 2. Materials and Methods

### 2.1. Study Participants

This was a prospective, randomized study. We recruited patients with a clinical diagnosis of COPD referred to the pulmonary rehabilitation program from the pulmonary outpatient department of the Tao-Yuan General Hospital, Ministry of Health and Welfare, Taiwan, if they met the following criteria: (1) diagnosed with COPD according to the Global Initiative for Chronic Obstructive Lung Disease (GOLD) criteria [13]; (2) in a medically stable condition (i.e., at least four weeks since the last exacerbation), (3) able to walk unassisted, and (4) ≥40 years of age. The exclusion criteria were receiving home oxygen therapy, hemodynamic instability, other pulmonary diseases, and comorbidities that affect physical activities in daily life such as severe neurological, musculoskeletal, or cardiovascular conditions. The study was performed in accordance with the Declaration of Helsinki and was approved by the hospital’s institutional review board (TYGH106081). Written informed consent was obtained from all patients prior to inclusion. Basic data such as demographics, anthropometrics, and diagnoses were recorded at the time of admission to the study.

### 2.2. Study Design

The subjects were randomly assigned to the intervention or control group according to a computer-generated algorithm. Subjects were randomly assigned to either the pedometer group (PG) or control group (CON).

In the pedometer group, patients received a pedometer and the task of walking at home for six weeks. They attached a pedometer (which shows the number of steps) on their wrist for approximately 12 h (from awakening until going to bed) each day, seven days/week for six weeks. They were also asked to walk at home as much as possible to try to reach the target daily steps. The target steps were set as 100–110% of their average daily steps from the previous week, with the first week’s target derived from the baseline measurement, eventually up to 10,000 steps. The investigators asked for patients’ subjective responses and checked the patients’ pedometers during weekly visits. The investigators then reset the target step for the following week according to the patients’ responses and pedometer daily step count. In the control group, patients received counseling during weekly visits for six weeks. Patients were encouraged to be active at home and walk ≥ 30 min per day without any supervision. Both groups received the same standard medical care from their chest physician, which consisted of standard monitoring and self-referral consultation if any symptoms worsened.

### 2.3. Measurements

At the beginning and end of the study, the following were assessed: 

Pulmonary function was assessed as forced vital capacity (FVC), forced expiratory volume in one second (FEV1), FEV1/FVC, and % FEV1 measured by a spirometer following the method reported by the ATS recommendations [14]. 

The subjective sensation of breathlessness was assessed using the modified Medical Research Council (mMRC) scale. The mMRC dyspnea scale contains five statements that patients rate on a scale of 0–4 reflecting minimal (e.g., “I only get breathless with strenuous exertion”) to severe symptoms (e.g., “I am too breathless to leave the house”) [15].

Exercise capacity was measured by a 6 min walking test and expressed as the 6 min walk distance (6 MWD). The test was performed by a well-trained respiratory therapist according to the ATS recommendations [16].

The level of physical activity was assessed as the number of daily steps, defined as the average step count of seven days obtained by the pedometer. At a week before and after completion of the study, all subjects were asked to wear wore the pedometer for seven continuous days. The investigators them collected pedometer and recorded the average daily steps from the pedometer data.

Quality of life status was assessed using the COPD Assessment Test (CAT) and short form 12 (SF-12) questionnaires. The CAT questionnaire is a disease-specific quality of life questionnaire. It has eight items related to symptoms, energy, sleep, and activity. According to the CAT, the degree of impact from diseases can be classified into four levels: slight impact (score 0–10), medium impact (score 11–20), high impact (score 21–30), and very high impact (score > 30) [17]. The SF-12 questionnaire is a short version of the Short Form 36 questionnaire, which is a generic quality of life questionnaire and has been applied to both healthy populations and patients with chronic diseases. The SF-12 questionnaire contains 12 items on two scales: physical (PCS) and mental health component scale (MCS) [12]. The SF-12 explains the majority of the variance (80–85%) of the SF-36. The outcomes range from 0 (worst conceivable QOL) to 100 (best conceivable QOL) [18].

### 2.4. Statistics

The primary outcome of the study was a change in daily step count, an indicator of PA. The sample size was calculated based on observations from a previous study, assuming a mean difference in daily step count of 725 steps per day between the groups [19]. A sample size of 31 subjects per group would be needed, yielding an analysis power of 80%, setting α to 0.05. The analysis was conducted using SPSS v.18 (SPSS, Chicago, IL, USA). The Shapiro–Wilk test was used to examine the normality of distribution. The results were expressed as the mean ± SD for nominal distributions and as median and interquartile range. (25–75 percentiles) for nonparametric distributions. Student’s t-test or Mann–Whitney U test was used when appropriate to examine the baseline differences between groups and paired t-test or Wilcoxon signed-rank test was used to examine within-group differences. Statistical significance was set at *p* < 0.05.

## 3. Results

**Figure 1:**
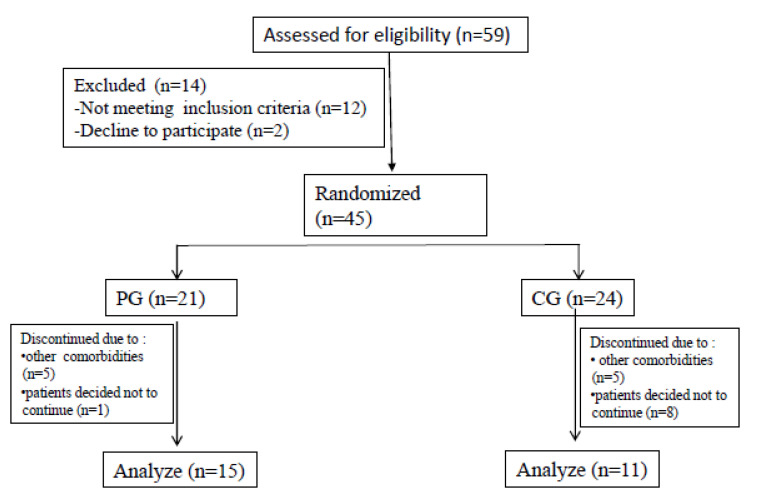
Flow chart of subject participation and analysis.

Fifty-nine consecutive eligible patients were screened from March 2018 to March 2019 (Figure 1). Fourteen patients were excluded because they did not meet the inclusion criteria (*n* = 14). Thus, 45 subjects were randomized into both the pedometer group (PG, *n* = 21) and control group (CG, *n* = 24). During the study period, six subjects in the PG and 13 subjects in the CG dropped out of the study due to comorbidities (musculoskeletal, cardiovascular, and the common cold) or lack of motivation, leaving 15 subjects in the PG and 11 subjects in the CG for analysis (Figure 1).

Table 1 presents a summary of the demographic and clinical characteristics of the participants. No significant differences existed between the groups in terms of age (73.5 ± 8.2 vs. 71.9 ± 11.1 years, *p* = 0.678), and BMI (0.2 ± 4.4 vs. 1.0 ± 2.3 kg/m^2^, *p* = 0.443). The majority of subjects in both groups were diagnosed with COPD GOLD stage II (53.3% in PG and 63.6% in CG). There were no significant differences in the baseline characteristics between the PG and CG except the % of predicted 6 min walking distance.

**Figure 2:**
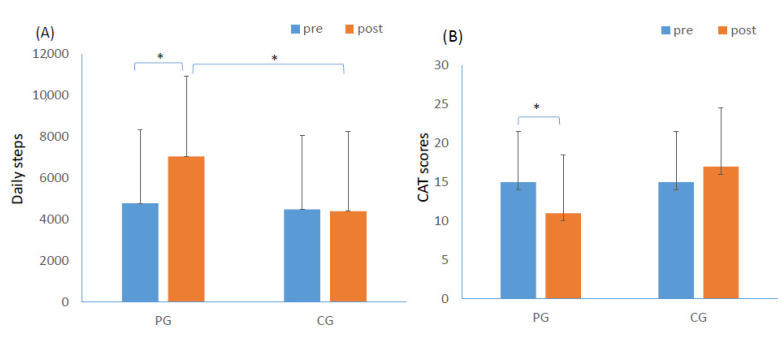
Comparisons of daily steps (**A**) and CAT scores (**B**) within subjects in PG and CG. *: *p* < 0.05.

In the PG, there was a significant improvement in pulmonary function, daily steps, dyspnea scale, and quality of life at the end of the study. The predicted % of FVC significantly increased from 81.0 ± 14.3% to 90.6 ± 23.4% (*p* = 0.021) (Table 2). The daily steps increased from 4768.4 ± 2643.3 steps to 7042.7 ± 4281.9 steps (*p* = 0.01) (Figure 2A). Post-CAT scores (11.5 ± 7.5) were significantly lower than those in the pre-CAT scores (14.9 ± 8.8) (Figure 2B). In the control group, there were no significant differences in the measurement of pulmonary function, 6 min walking distance, dyspnea scale, and quality of life score between pre-and post-measurements. The number of daily steps decreased from 4468.08 ± 3783.9 steps to 4385.1 ± 3692.6 steps (*p* > 0.05) (Figure 2A).

After the study, subjects in the PG showed a significant improvement in the daily steps (2274.3 ± 3014.2 steps) compared with those in the CG (−83.68 ± 923.30 steps) (*p* = 0.011) (Table 3). There was also a significant difference in the changes in CAT scores between the PG (−4.80 ± 6.6) and CG (1.55 ± 6.5) (*p* = 0.022) (Table 3). No significant difference was found in the changes in pulmonary function, 6 min walking distance, and dyspnea scale between the PG and CG at the end of the study (Table 3).

The relationship between changes in the parameters is presented in Table 4. Changes in daily steps were significantly correlated with changes in the MRC scale (*r* = 0.385, *p* = 0.047) and CAT scores (*r* = 0.505, *p* = 0.010). A greater increase in daily steps was associated with greater improvement in the dyspnea scale and symptom-related quality of life questionnaire scores.

## 4. Discussion

The main finding of this randomized controlled trial demonstrated that in patients with COPD, a step target using a pedometer-based home program produced a significant increase in daily steps. This daily walking program also resulted in improvement of pulmonary function and perception of health status. However, no significant improvement was observed in exercise capacity.

In our study, subjects in the PG had significantly increases in FVC and FVC% after the intervention, whereas subjects in CG showed no significant differences (Table 2). In addition, although it was not statistically significant, the changes in % predicted FEV1 in PG showed a tendency of increase, whereas a tendency of decrease was found in CG (Table 2 and Table 3). In a study examining the effects of a self-monitored, home-based exercise training program on patients with moderate COPD, subjects in the intervention group demonstrated a significant improvement in FEV1%, whereas subjects in the control group showed a tendency of decrease in FEV1% after the 3-month program [20]. A meta-analysis of 21 randomized, controlled trials reported that exercise training was associated with a small but significant increase in spirometry results (e.g., FEV1, FVC) [21]. During walking, regular and frequent body movements induce deep breathing, enhance chest expansion, increase pulmonary ventilation, and may result in the improvement of pulmonary function. The results of our study are consistent with those of a previous study that showed that physical activity is associated with the improvement or maintenance of lung function in COPD patients.

The level of physical activity is often decreased in patients with COPD due to exertion dyspnea and exercise intolerance, leading to further deconditioning. In our study, the daily steps in the PG significantly increased from 4768.4 ± 2643.3 steps/day to 7042.7 ± 4281.9 steps/day after a 6-week intervention. The improvement in daily steps in the PG was also significantly higher than that in the CG. Mendoza et al. evaluated the effects of a 3-month pedometer-based program on the level of physical activity in patients with COPD [22]. They found that the PG had significantly greater improvement in daily steps than the patients in the control group [22]. Kawagoshi et al. demonstrated a significantly higher daily walking time in COPD patients who participated in a home-based pedometer program than in the CG [23]. The commercialized pedometer, which provides immediate visual feedback, may increase subjects’ motivation and compliance to accomplish the set goal. With a pedometer, subjects become aware of their current number of steps and increase their efforts to change their behavior [24]. Our results appear to support previous findings which suggest the benefits of pedometer-based programs on the improvement in the level of physical activities.

The effects of a pedometer-based program among COPD patients have been proposed in previous studies. Moy et al. set the goal of daily steps to be an increase of 600 steps/week and reported an improvement of 447 steps/day (about 13%) after a 16-week home program [25]. In another study, the goal of daily steps was set to increase 15%/month in the experimental group and the subjects have a mean change in 1114 steps (36%) after a 12-week intervention [26]. Compared to previous studies [25,26], our study achieved a higher increase of daily steps (2274 steps, about 47%) with a shorter duration (6 weeks). Physiological capacity is increased by physical training stimulus on a regular basis. To build a new adaptation, the training stimulus must be increased to maintain overload [27]. The prescription of the overloading dose is also essential for the improvement of capacity. Either too small or too high overloading dose could lead to no improvement or, even worth, tissue damage. In our study, the target of daily steps was set individually (an increase of 10% of the subject’s average daily steps from the previous week) which may be more precisely fit patients’ capacity and lead to improvement of daily steps with better efficiency.

In our study, quality of life was assessed using CAT and SF12 questionnaires. The patients in the PG demonstrated significant improvements in CAT scores after the intervention program. The changes in CAT levels in the PG were significantly higher than those in the CG. In addition, an increase in the number of daily steps was associated with improvement in dyspnea level (mMRC scale) and quality of life (CAT scores). Widyastutia et al. reported that COPD patients demonstrated a significant improvement in daily steps and mMRC and CAT scores after six weeks of a home pedometer-based walking program [28]. In a study examining the effects of a pedometer-based program on COPD patients, Mendoza et al. found that the experimental group showed increased improvement in daily steps and CAT scores [22]. The results of our study are consistent with those previous studies and suggested that a pedometer-based program can improve the level of daily activity and quality of life in COPD patients. Regular and sustained participation in physical activity (PA) can assist in the prevention and/or slow down the progress of several chronic diseases in relation to both primary and secondary prevention [29]. PA improves peripheral muscle function in patients with COPD [28]. It also reduces the number of hospital admissions, which have been shown to impair QoL [30]. The results of our study were consistent with those of previous studies that showed that COPD patients who maintain or increase their levels of daily activity may break the vicious cycle of inactivity and improve their QoL.

A pulmonary rehabilitation program that includes exercise has been recommended for patients with COPD to improve level of physical activity and quality of life [8]. Many traditional pulmonary rehabilitation programs are held in hospitals and could be difficult for patients to access considering the transportation requirements and distance from the hospitals. Over the last two years, the world has been consumed by the COVID-19 pandemic. Despite great efforts to control the pandemic, the infection rates are still high and the healthcare system face enormous challenge around the world. Barriers for patients with COPD to effective pulmonary rehabilitation existed prior to the COVID-19 pandemic and were exacerbated by the pandemic because of the need for social distancing, widespread lockdowns, and overload on health care systems. The pedometer-based program in our study can be followed at home and thus may lower the risk of contagion during the transportation to hospital. This program may be considered to be an alternative program for patients to maintain their physical function during epidemic conditions.

In a previous study, COPD patients with a mean disease severity of GOLD level 2.2 demonstrated significant improvement in 6 min walking distance (6 MWD) from 404 ± 148 m to 467 ± 157 m after participating in a walking program with a pedometer for feedback [23]. However, in our study, no significant changes in 6 MWD (450.5 ± 102.4 m vs. 450.0 ± 108.4 m) were found after the intervention program in the PG. This could be due to the ceiling effect as the majority of subjects were classified as GOLD II. Spruit et al. performed 6 min walking test for thousands of COPD patients and reported that the mean 6 MWD was 409 ± 112 m [31]. In our study, the mean 6 MWD of the PG at baseline was 450.5 ± 102.4 m, which was much higher than that in the previous study. The % of predicted 6 MWD at baseline (115.2 ± 25.9%) was already over 100% in the PG, which may result in the ceiling effect and thus may not be able to gain additional improvement in the performance of 6 min walking test.

### Limitation

This study had several limitations. First, the self-efficacy of the subjects in this study was not evaluated. Second, subjects were not blinded to their group, which may have influenced the motivation of the subjects in the CG. However, both groups received the same amount of face-to-face contact, meaning that differences between groups were not due to different levels of input from health professionals but rather were related to the specific nature of the intervention. Third, the small sample size and high number of drop off in our study may have affected the statistical power of detecting the differences of the results. The small sample size may also limit the representative ability of COPD. Further studies with larger sample sizes should be carried out.

## 5. Conclusions

In conclusion, the study findings indicate that the pedometer-based, step target home program enhances the quality of life and the level of physical activity by increasing daily steps in patients with COPD. These novel data provide significant insights for developing an appropriate home rehabilitation program for COPD patients by healthcare providers with the assistance of commercial devices such as pedometers. Overall, our results support the use of specific programs that include the use of monitoring devices and goal setting to promote positive outcomes in patients with COPD.

## Figures and Tables

**Table 1:** Demographic data of subjects in pedometer (PG) and control group (CG).

	PG (*n* = 15)	CG (*n* = 11)	*p*
Gold *n* (%)			0.70
II	8 (53.3%)	7 (63.6%)	
III	7 (46.7%)	4 (36.4%)	
Gender			1.00
Male	13 (86.7%)	9 (81.8%)	
Female	2 (13.3%)	2 (18.2%)	
Age (year)	73.5 ± 8.2	71.9 ± 11.1	0.678
Body height (cm)	163.5 ± 6.8	163.0 ± 9.2	0.881
Body weight (kg)	59.0 ± 10.9	56.2 ± 10.0	0.512
BMI (kg/m^2^)	22.2 ± 4.4	21.0 ± 2.3	0.443
Pulmonary function test			
FEV1 (L)	1.2 ± 0.4	1.3 ± 0.6	0.718
Predicted FEV1(%)	51.2 ± 13.7	53.0 ± 16.3	0.750
FVC (L)	2.5 ± 0.7	2.8 ± 1.1	0.299
Predicted FVC(%)	81.0±14.3	84.3 ± 32.5	0.751
FEV1/FVC (%)	49.8 ± 15.2	47.7 ± 19.0	0.762
mMRC score	1.5 ± 1.1	1.6 ± 0.7	0.829
Exercise capacity			
6 MWD(m)	450.5 ± 102.4	397.8 ± 110.9	0.153
% of predicted 6 MWD	115.2 ± 25.9%	74.5 ± 37.5%	0.014
Daily steps (steps)	4768.4 ± 2643.3	4468.8 ± 3783.9	0.700
Quality of life			
SF12 PCS	35.5 ± 10.2	38.2 ± 7.1	0.608
SF12 MCS	48.7 ± 10.9	46.8 ± 10.3	0.550

**Table 2:** Comparisons of outcomes within pedometer (PG) and control group (CG).

	PG			CG		
	Pre	Post	*p*	Pre	Post	*p*
Pulmonary function						
FEV1 (L)	1.2 ± 0.4	1.2 ± 0.4	0.181	1.3 ± 0.6	1.3 ± 0.6	0.859
Predicted FEV1(%)	51.2 ± 13.7	53.7 ± 15.9	0.222	53.0 ± 16.3	51.6 ± 16.1	0.831
FVC (L)	2.5 ± 0.7	2.8 ± 0.9	0.022 *	2.8 ± 1.1	2.9 ± 1.1	0.823
Predicted FVC (%)	81.0 ± 14.3	90.6 ± 23.4	0.021 *	84.3 ± 32.5	90.5 ± 24.6	0.439
FEV1/FVC (%)	49.8 ± 15.2	47.5 ± 15.9	0.210	47.7 ± 19.0	47.6 ± 20.1	0.891
Dyspnea						
mMRC	1.5 ± 1.1	1.6 ± 1.1	0.50	1.6 ± 0.7	1.8 ± 0.9	0.192
Exercise capacity						
6 MWD (m)	450.5 ± 102.4	450.0 ± 108.4	0.969	397.8 ± 110.9	412.2 ± 108.8	0.212
% of predicted 6 MWD	115.2 ± 25.9%	114.6 ± 26.6%	0.815	74.5 ± 37.5%	74.4 ± 38.1%	0.992
Quality of life						
SF12 PCS	35.5 ± 10.2	38.4 ± 9.8	0.189	38.2 ± 7.1	41.1 ± 10.3	0.168
SF12 MCS	48.7 ± 10.9	50.8 ± 10.3	0.310	46.8 ± 10.3	46.9 ± 12.0	0.991

6 MWD: 6 min walking distance. SF12 PCS: Short form 12 Physiologic component section. SF12 PCS: Short form 12 Mental component section. *: *p* < 0.05.

**Table 3:** Comparison of changes in parameters between pedometer (PG) and control group (CG).

	PG	CG	*p*
ΔFEV1 (L)	0.06 ± 0.16	−0.01 ± 0.17	0.310
ΔPredicted FEV1 (%)	2.55 ± 7.6	−1.44 ± 7.3	0.192
ΔFVC (L)	0.28 ± 0.40	0.02 ± 0.32	0.081
ΔPredicted FVC (%)	9.67 ± 14.3	6.15 ± 25.1	0.650
ΔFEV1/FVC (%)	−2.26 ± 6.7	−0.09 ± 2.3	0.323
ΔDyspnea scale-mMRC	0.13 ± 0.74	0.21 ± 0.58	0.745
Δ6 MWD (meter)	−0.53 ± 50.0	12.6 ± 39.4	0.481
ΔDaily steps (steps)	2274.3 ± 3014.2	−83.7 ± 923.3	0.011 *
ΔCAT	−4.80 ± 6.06	1.55 ± 6.5	0.022 *
ΔSF12 PCS	2.96 ± 8.0	3.51 ± 6.4	0.859
ΔSF12 MCS	2.1 ± 7.4	−0.05 ± 14.1	0.629

6 MWD: 6 min walking distance. SF12 PCS: Short form 12 Phyisologic component section. SF12 PCS. Short form 12 Mental component section. *: *p* < 0.05.

**Table 4:** The correlation between changes of parameters in all subjects.

	ΔDaily Steps	ΔCAT
ΔFEV1 (L)	0.073	0.399
ΔPredicted FEV1 (%)	0.200	0.439 *
ΔFVC (L)	0.287	0.356
ΔPredicted FVC (%)	0.180	0.289
ΔFEV1/FVC (%)	0.096	0.053
ΔmMRC	0.385 *	0.405
Δ6 MWD (meter)	0.380	0.022
ΔDaily steps	1	0.505 *

*: *p* < 0.05.

## Data Availability

All data will be available from the corresponding author on reasonable request.
